# Screening of *Ipomoea tuba* Leaf Extract for Identification of Bioactive Compounds and Evaluation of Its *in vitro* Antiproliferative Activity Against MCF-7 and HeLa Cells

**DOI:** 10.17113/ftb.58.01.20.6351

**Published:** 2020-03

**Authors:** Thirupati Chinna Venkateswarulu, Gaddam Eswaraiah, Srirama Krupanidhi, Karlapudi Abraham Peele, Indira Mikkili, Alugunulla Venkata Narayana, Bharath Kumar Ravuru, John Babu Dulla, Ranga Rao Ambati

**Affiliations:** 1 Department of Bio-Technology, Vignan’s Foundation for Science, Technology & Research (Deemed to be University), Vadlamudi-522213, Guntur, Andhra Pradesh, India; 2 School of Life Sciences, Rayalaseema University, Kurnool-518002, Andhra Pradesh, India

**Keywords:** mangrove species, *Ipomoea tuba*, bioactive compounds, antiproliferative activity

## Abstract

Mangroves contain a wide range of bioactive compounds with pharmacological activities. In the present study, we analysed the separation and detection of phytoconstituents with the methanol extract of *Ipomoea tuba* leaf using gas chromatography-mass spectrometry (GC-MS) and tested its *in vitro* cytotoxicity effect against MCF-7 and HeLa cells. Phytochemical compounds such as docosanoic, octadecatrienoic and *cis*-9-octadecanoic acids, triterpenoid γ-sitosterol, and terpene alcohol in methanol extract of *I. tuba* leaf were identified. Furthermore, *in vitro* antiproliferative activity of the extract of *I. tuba* leaf was evaluated using MCF-7 and HeLa cells. The results indicated a reduction of cell viability of 37.43 and 41.89% of MCF-7 and HeLa cells respectively. The methanol extract of *I. tuba* leaf proved to be effective in protecting the cells against oxidative stress. This is the first report on the *in vitro* cytotoxicity effect of *I. tuba* leaf extract on MCF-7 and HeLa cells.

## INTRODUCTION

Mangrove ecosystem plays a major role in the human life to protect us from natural disasters like tsunami, floods, high tides and soil erosion. Mangrove plants grow in water logging region and offer a shelter for wide ranges of endemic fauna and flora (*1*, *2*). They absorb and remove five times more carbon dioxide than normal terrestrial plants (*3*). The mangrove species, namely *Suaeda maritima,* commonly known as seablite is used in homemade foods such as salad, curry, soy sauce, and spicy soup in Thailand (*4*). In recent years, bioactive compounds produced from the plants have attracted the interest of pharmaceutical industries for formulation of drugs because the effectiveness of synthetic antibiotics against several pathogenic strains is slowly decreasing (*5*). The natural compounds and related drugs are used to treat different human diseases (*6*). The crude methanol extract of different medicinal plants contains many bioactive compounds having anticancer activity against several cancers like gastric, colon and breast cancer cell lines (*7*). Phytoconstituents and cytotoxicity of *I. tuba* have not been studied so far. Hence, the present study aims to evaluate the phytoconstituents of methanol extract of *I. tuba* leaf and their cytotoxicity effects on MCF-7 and HeLa cells.

## MATERIALS AND METHODS

### Sample preparation

*Ipomoea tuba* sample was collected from Nizampatnam mangroves, Guntur, Andhra Pradesh, India. The sample was prepared from leaves of *I. tuba* by soaking 50 g of powdered sample in 50 mL of absolute methanol for 72 h. The sample was filtered through Whatman No. 42 filter paper and then methanol was evaporated from the test sample by rotary vacuum evaporator (EV11, Equitron Medica Pvt Ltd, Mumbai, India). The final crude extract was dissolved in 100% dimethyl sulfoxide (DMSO; Sigma-Aldrich Chemicals Pvt Ltd, Merck, Bangalore, India), made to final concentration of 100 mg/mL and used for antiproliferative studies. The concentration of DMSO maintained in the wells was less than 1%, which is not toxic to the cell lines (*8*, *9*).

### Identification of compounds by GC-MS analysis

Bioactive compounds in leaf extract of *I. tuba* were identified by GC-MS (6890 series; Agilent, Santa Clara, CA, USA). The following chromatographic conditions were maintained: initial column temperature 30 °C, heated up to 300 °C at 10 °C/5 min, flow rate 1.0 mL/min and helium was used as carrier gas in split mode. The bioactive compounds were identified based on retention times and quantified by integration of peak area. Similarity of compounds was compared with known compounds using NIST based AMDIS software (*10*).

### 3-(4,5-dimethylthiazol-2-yl)-2,5-diphenyltetrazolium bromide (MTT) assay for cell viability

*Ipomoea tuba* leaf extract is screened for *in vitro* cytotoxicity activity on MCF-7 and HeLa cells (5x10^3^ cell/well) using MTT (Sigma-Aldrich, Merck, St. Louis, MO, USA) assay. The sample (100 µL diluted plant extract) was added to 100 µL of Dulbecco`s Modified Eagle’s medium (DMEM), then the cell lines were added to the 96-well microtiter plate, and incubated for 48 h at 37 °C. The MTT was added and allowed to incubate for 2 h until the purple precipitate was formed. Then, absorbance values were measured at 520 nm using UV-Vis spectrophotometer (Cary 60; Agilent Technologies, Selangor Darul Ehsan, Malaysia). The dose-response curve was plotted for evaluation of IC_50_ values (*11*).

### Statistical analysis

The experimental data of both cell lines were statistically analyzed using ANOVA method. The value p*<*0.05 is considered statistically significant for the analysis of the percentage of inhibition of cell viability.

## RESULTS AND DISCUSSION

### GC-MS analysis for compound identification

The chromatogram confirmed the presence of compounds such as fatty acids: docosanoic, octadecatrienoic and *cis*-9-octadecanoic acids, triterpenoid γ-sitosterol, and terpene alcohol in the leaf extract of mangrove plant *Ipomoea tuba*. The compounds were identified based on retention times. Table 1 gives the molecular mass and retention times of each compound. Angaye *et al.* (*12*) reported various bioactive compounds in the extracts of mangroves *Rhizophora mangle, Rhizophora racemosa*, *Avicennia germinans* and *Laguncularia racemosa*. Phytochemical compounds such as β-sitosterol, eicosanol and taraxerol are found in *Bruguiera cylindrica* extract and showed their cytotoxicity against neuro2A cancer cell lines (*13*).

**Table 1:** Bioactive compounds identified in the extract of *Ipomoea tuba* leaf by GC-MS

Peak no.	*t*_R_/min	Compound name	Formula	*M*/(g/mol)	CAS number
1	19.49	docosanoic acid	C_21_H_44_COOH	340.59	112-85-6
2	21.24	3,7,11,15-tetramethyl-2-hexadecene-1-ol	C_20_H_40_O	296.00	7541-49-3
3	27.86	octadecatrienoic acid-ethyl ester	C_19_H_32_O	292.46	1191-41-9
4	31.07	*cis*-9-octadecanoic acid	C_18_H_34_O_2_	282.00	112-80-1
5	32.80	γ-sitosterol	C_29_H_50_O	414.71	83-47-6

The bioactive compounds from *Avicennia marina, Salvadora persica* and *Avicennia officinalis* contain several types of secondary metabolites like flavonoids, tannins, alkaloids and saponins (*14*, *15*). In previous study, GC-MS analysis of the extracts of *Acrostichum aureum* confirmed the presence of bioactive compounds such as stigmasterol, γ-sitosterol, campesterol and 24-methylene cycloartenol with potential activity against adenocarcinoma, carcinoma and other human cancer cell lines (*16*). Ganesh and Vennila (*17*) reported the presence of different bioactive compounds, namely terpenoids, steroids, saponins, catechol and phenols in the methanol extracts of *Acanthus ilicifolius* and *Avicennia officinalis.* Report from Basyuni *et al.* (*18*) showed that the mangrove species *Acanthus ilicifolius, Rhizophora apiculata, Sonneratia caseolaris* contain wide range of phytoconstituents like triterpenoids, taraxerol, germanicol and tannins, with anti-inflammatory, anticarcinogenic, antimicrobial and antiprotozoan activities. The mangrove plant *Rhizophora mucronata* leaf extract contains vindoline, catharanthine and serpentine, the major alkaloids with free radical scavenging and cytotoxicity activity (*19*). The findings of Grozav *et al*. (*20*) proved that the derivative of thiazole synthesized from mangrove plant leaves has potential anticancer effect against ovarian cancer cell lines A2780 and HeLa cell lines.

### In vitro antiproliferative activity of I. tuba leaf extract on MCF-7 cells

**Fig. 1:**
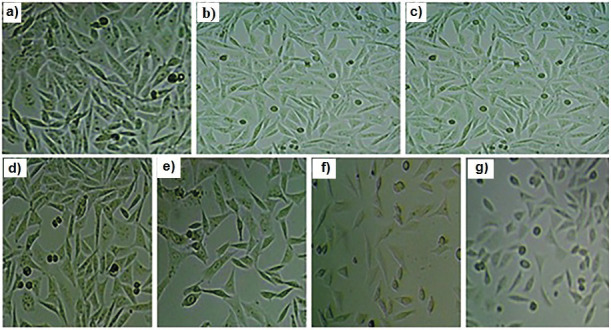
Morphology of MCF-7 cells after the treatment with the extract of *Ipomoea tuba* leaf: a) untreated MCF-7 cell lines, and b-g) treated with different concentrations (5, 10, 25, 50, 75 and 100 µg/mL respectively) of *I. tuba* leaf extract

Extract of *I. tuba* leaves showed significant *in vitro* antiproliferative effect on MCF-7, and the viability of MCF-7 was reduced with the increase in the concentration of the sample. High reduction of MCF-7 cells was observed at the concentration of *I. tuba* leaf extract of 100 µg/mL (Table 2) and the IC_50_ value against MCF-7 cells was found to be (40.4±0.1) μg/mL. During experiments, it was observed that the increase in sample concentration altered the morphology of MCF-7 cells, leading to cell death (Fig. 1)^. Similar findings are reported for^ *^in vitro^* ^cytotoxicity effect of^ *^Avicennia marina^* ^extracts on different cancerous cells (^*^21^**^-^**^23^*^)^. Patra and Thatoi (*24*) reported the antiproliferative activity of the methanol extract of *Heritiera fomes* leaves against melanoma cell lines and achieved 40% inhibition. The methanol extract of *Avicennia marina* leaf showed anticancer activity against MDA-MB 231 and MCF-7 cell (*25*).

**Table 2:** Inhibition of MCF-7 and HeLa cells using *Ipomoea tuba* leaf extract

*γ*(*I. tuba* leaf extract)/(μg/mL)	Viability of MCF-7 cells/%	Viability of HeLa cells/%
100	(37.4±0.1)^f^	(41.4±0.1)^f^
75	(42.0±0.2)^e^	(44.7±0.2)^e^
50	(45.4±0.6)^d^	(48.3±1.0)^d^
25	(53.5±0.8)^c^	(51.4±0.4)^c^
10	(55.8±0.5)^b^	(53.9±0.3)^b^
5	(60.7±0.3)^a^	(55.6±0.8)^a^

Values are expressed as mean±S.D. Values with different letters in superscript in the same column are significantly different (p<0.05) determined by ANOVA.

### In vitro antiproliferative activity of I. tuba leaf extracts on HeLa cells

**Fig. 2:**
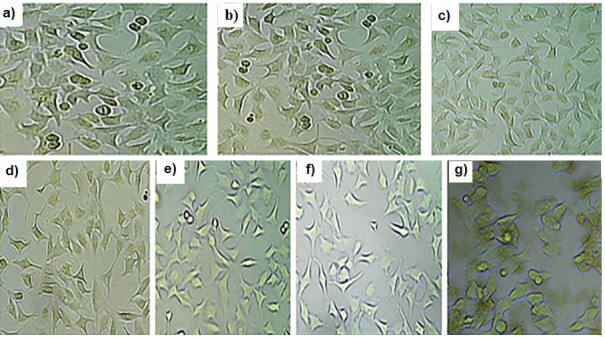
Morphology of HeLa cells after the treatment with the extract of *Ipomoea tuba* leaf: a) untreated HeLa cell lines, and b-g) treated with different concentrations (5, 10, 25, 50, 75, and 100 µg/mL) of *I. tuba* leaf extract

HeLa cell viability was decreased with the increased concentration of leaf extract and the maximum reduction in HeLa cells was observed at 100 µg/mL (Table 2), with the IC_50_ value of (39.4±0.1) μg/mL. It was observed that after the treatment with *I. tuba* extract, the HeLa cells slowly detached from one another. Fig. 2 shows the change in morphology of HeLa cells. Khajure and Rathod (*26*) reported that the extract of *A. ilicifolius* had cytotoxic activity against HeLa and KB cells. Rajeswari *et al*. (*27*) also reported that the flavone molecule from *Excoecaria agallocha* has the cytotoxic activity against HeLa cells.

## CONCLUSION

In conclusion, GC-MS analysis confirmed the presence of different phytoconstituents and the *Ipomoea tuba* extracts were proved to have antiproliferative effect on MCF-7 and HeLa cells. This is the first report of high antiproliferative activity of the extract of *I. tuba* leaf on MCF-7 and HeLa cells. Furthermore, these bioactive compounds could be used in functional food applications for health benefits.
